# Mechanism of Action, Resistance, Synergism, and Clinical Implications of Delamanid Against Multidrug-Resistant *Mycobacterium tuberculosis*

**DOI:** 10.3389/fmicb.2021.717045

**Published:** 2021-10-07

**Authors:** Saeed Khoshnood, Elahe Taki, Nourkhoda Sadeghifard, Vahab Hassan Kaviar, Mohammad Hossein Haddadi, Zahra Farshadzadeh, Ebrahim Kouhsari, Mehdi Goudarzi, Mohsen Heidary

**Affiliations:** ^1^Clinical Microbiology Research Center, Ilam University of Medical Sciences, Ilam, Iran; ^2^Department of Microbiology, School of Medicine, Tehran University of Medical Sciences, Tehran, Iran; ^3^Infectious and Tropical Diseases Research Center, Health Research Institute, Ahvaz Jundishapur University of Medical Sciences, Ahvaz, Iran; ^4^Department of Microbiology, School of Medicine, Ahvaz Jundishapur University of Medical Sciences, Ahvaz, Iran; ^5^Laboratory Sciences Research Center, Golestan University of Medical Sciences, Gorgan, Iran; ^6^Department of Microbiology, School of Medicine, Shahid Beheshti University of Medical Sciences, Tehran, Iran; ^7^Department of Laboratory Sciences, School of Paramedical Sciences, Sabzevar University of Medical Sciences, Sabzevar, Iran; ^8^Cellular and Molecular Research Center, Sabzevar University of Medical Sciences, Sabzevar, Iran

**Keywords:** *Mycobacterium tuberculosis*, tuberculosis, delamanid, review, TB

## Abstract

Multidrug-resistant (MDR) isolates of *Mycobacterium tuberculosis* (MTB) remain a primary global threat to the end of tuberculosis (TB) era. Delamanid (DLM) is a nitro-dihydro-imidazooxazole derivative utilized to treat MDR-TB. DLM has distinct mechanism of action, inhibiting methoxy- and keto-mycolic acid (MA) synthesis through the F420 coenzyme mycobacteria system and generating nitrous oxide. While DLM resistance among MTB strains is uncommon, there are increasing reports in Asia and Europe, and such resistance will prolong the treatment courses of patients infected with MDR-TB. In this review, we address the antimycobacterial properties of DLM, report the global prevalence of DLM resistance, discuss the synergism of DLM with other anti-TB drugs, and evaluate the documented clinical trials to provide new insights into the clinical use of this antibiotic.

## Introduction

Notwithstanding the use of potent agents in varying combination treatment regimens, *Mycobacterium tuberculosis* (MTB) has an independent ability to resist antitubercular drugs (Li et al., 2019). A proper pharmacological regimen can help bacteriological and clinical treatments and inhibit the emergence and spread of resistance. However, limited treatment options make the management of multidrug-resistant tuberculosis (MDR-TB) and other chronic TB cases clinically challenging, as well as raise public health concerns. Likewise, the lack of sufficient active drugs during the intensive and continuation phases of treatment not only hinders the survival of the patients but also induces further resistance (Borisov et al., 2017; Heidary et al., 2019).

Drug progress in TB therapeutics has evolved considerably with the introduction of two new drugs, namely, bedaquiline (BDQ) and delamanid (DLM). The European Medicines Agency conditionally approved DLM as a first-in-class bicyclic nitroimidazole, relying on promising results from phase IIb trial and with regard to medical need for treating MDR-TB (Blair and Scott, 2015; Khoshnood et al., 2021).

Based on the 2017 WHO Global TB Report, using BDQ and DLM was initiated in 89 and 54 countries, respectively (Cox et al., 2018). The rapid acquisition of resistance by DLM highlights the demand for the proper use of such new drugs and medication, and emphasizes the significance of drug resistance surveillance. Combination therapy with DLM and other active anti-TB agents is suggested for the prevention of acquired resistance (Diel et al., 2015).

This review aims to discuss the synergism of DLM with other antimycobacterial agents, to summarize the available evidence on DLM resistance among drug-resistant MTB isolates, and to evaluate the clinical use of this drug, in order to provide new insights into this phenomenon.

## Antimicrobial Properties

### Structure of Drug

**FIGURE 1:**
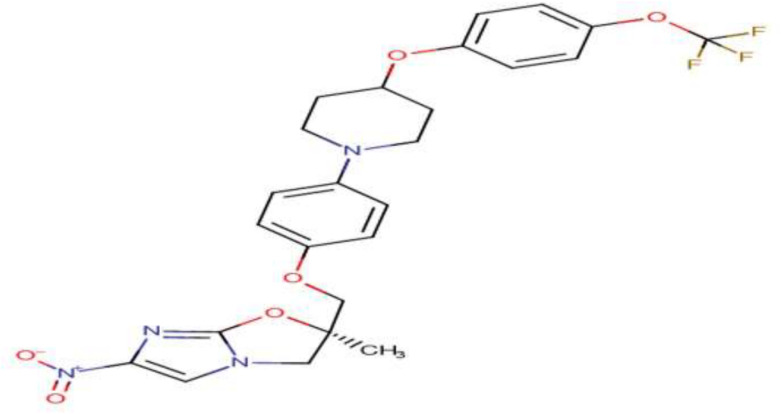
Chemical structure of delamanid (Szumowski and Lynch, 2015).

DLM, developed by Otsuka Pharmaceutical Co., Ltd. (Tokyo, Japan), belongs to a class of bicyclic nitroimidazole (Saliu et al., 2007; World Health Organization, 2020). It is one of the two new anti-TB drugs globally approved in the past 40 years (Ryan and Lo, 2014; von Groote-Bidlingmaier et al., 2019). Bicyclic nitroimidazooxazole, an analog of azomycin known as 2-nitro-imidazole, is isolated from *Streptomyces eurocidicus* and has potent *in vitro* and *in vivo* anti-TB activity (Igarashi, 2017). Substituents in the 2-position of 6-nitro-2,3-dihydroimidazo[2,1-b]oxazole accelerated anti-TB activity and declined mutagenicity. However, substitution in the 2-position of the side chain with a heteroatom eliminated mutagenicity (Figure 1). The 6-nitro-2,3-dihydroimidazo[2,1-b]oxazole is a racemic mixture so that right-handed, but not left-handed, enantiomers have activity against *Mycobacterium tuberculosis* complex (MTBC). The 6-nitro-2,3-dihydro-imidazooxazol (C_25_H_25_F_3_N_4_O_6_), commercially named Deltyba, was introduced as a safe compound with *in vivo* and *in vitro* optimal performance (Sasaki et al., 2006; Matsumoto et al., 2007; Mukherjee and Boshoff, 2011; Tsubouchi et al., 2016).

### Mechanism of Action

**FIGURE 2:**
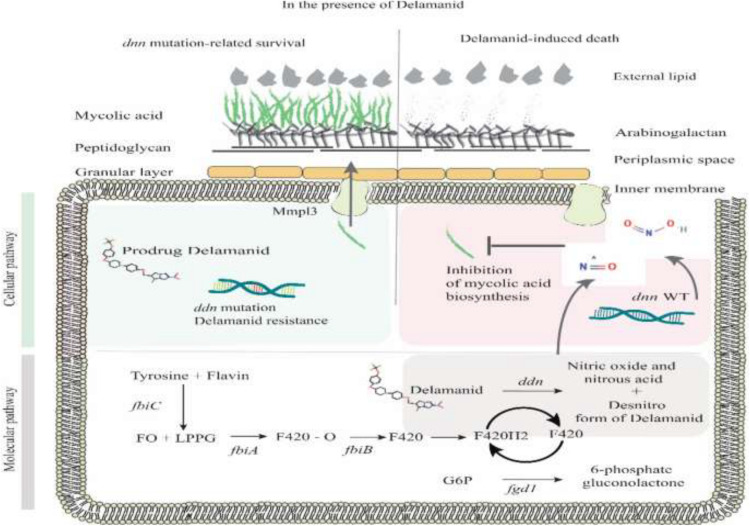
Mechanism of delamanid resistance.

Delamanid (DLM) is a prodrug that confers mycobactericidal activity by inhibiting the synthesis of methoxy and keto MA through the mycobacteria F420 system and generating nitrous oxide (Singh et al., 2008; Vilchèze, 2020). Both DLM and isoniazid (INH) act by preventing the synthesis of MA, which plays a pivotal role in the survival of mycobacterial genus. Compared with DLM, INH, as the inhibitor of α-MA synthesis, has a different strategy for impeding the cell wall synthesis (Lewis and Sloan, 2015). Enoyl-acyl carrier protein reductase, *InhA*, is a specific factor for the function of INH, while DLM needs mycobacterial F420 system for its activation (Parikh et al., 2000; Deane and Porkess, 2018). This system is the analog of flavin mononucleotide complex and composed of two enzymes, deazaflavin-dependent nitroreductase (Ddn, Rv3547) and F420-dependent glucose-6-phosphate dehydrogenase (G6PD; FGD1, Rv0407), as well as four coenzymes, *Fbi*A (Rv3361), *Fbi*B (Rv3261), *Fbi*C (Rv1173), and Rv0132c (Bashiri et al., 2010; Hartkoorn et al., 2014). All of these genes and coenzymes are involved in the synthesis and recycling of cofactor F-420. As depicted in Figure 2, DLM has undergone the influence of the Ddn enzyme for converting into its active and inactive forms, an unknown reactive intermediate metabolite that is active against MTB and a desnitro (inactive) form, respectively. The main function of DLM in preventing MA biosynthesis is attributed to the reactive intermediate metabolite. The removal of this major compound from the Mycobacterium cell wall leads to the destruction of this bacterium. G6PD is also responsible for returning the F420 to the reduced form (Gurumurthy et al., 2012; Greening et al., 2016; Schuster Bruce et al., 2019).

### Spectrum of Activity

#### Activity Against Mycobacterium Tuberculosis

Delamanid (DLM) has *in vitro* potential activity against standard, clinical susceptible and resistant strains of MDR-TB and also against extensive drug-resistant tuberculosis (XDR-TB) (Stinson et al., 2016). This drug is a narrow-spectrum antibiotic able to eliminate only MTBC, and its activity against non-tuberculosis mycobacteria (NTM) is unknown. Unpublished data from an earlier research has portrayed that DLM has no activity against aerobic and anaerobic intestinal microflora (Liu et al., 2018). However, another survey reflected that DLM affects the visceral leishmaniasis caused by *Leishmania donovani* (Patterson et al., 2016). Due to the lack of cross-resistance and antagonistic activity with other drugs, including INH, rifampin (RIF), and ethambutol (ETB), DLM has been suggested to treat TB (Parikh et al., 2000; Lewis and Sloan, 2015). Based on the fractional inhibitory concentration index (FICI), the aforesaid drugs are classified as synergistic or partially synergistic for tested strains (Matsumoto et al., 2006).

Delamanid (DLM) shows dose-dependent bactericidal activity in murine and guinea pig models infected by MTB (Chen et al., 2017). Similar to RIF (3 μg/ml), DLM (0.1 μg/ml at 4 h) has a dose-dependent activity against intracellular mycobacteria in human macrophage. The required dose of DLM to reduce 95% of colony-forming unit (CFU) was 0.625 mg/kg. This dose is 5.6 to 256 times less than the dose needed for first-line drugs, such as RIF, INH, and ETB, used in the treatment of MTB (Matsumoto et al., 2006). Early bactericidal activity (EBA) of DLM (in four doses: 100, 200, 300, and 400 mg daily) was assessed by Diacon et al. in 48 patients with pulmonary TB. The reported mean of the EBA of DLM in all doses was relatively low (0.04 log_10_ CFU/m). The pharmacokinetic (PK) assay in that study revealed that exposure to DLM did not increase in proportion to the drug dose and plateaued at a dosage of 300 mg (Diacon et al., 2011). Stinson and associates reported a very low minimum inhibitory concentration (MIC; 0.001–0.05 μg/ml) of DLM against the clinical and reference strains of MTB (Stinson et al., 2016). However, MTB mutant strains with a mutation in each of the five genes of the F420-dependent pathway developed resistance against DLM (MIC > 8 μg/ml) (Fujiwara et al., 2018; Jing et al., 2019; Polsfuss et al., 2019). Delamanid is accumulated in the cell and has pharmacological potential activity against the replicating, dormant, and intra- and extracellular bacilli (Chen et al., 2017). Gler and colleagues reported the results of the phase II trial (trial 204) on 481 patients infected with pulmonary MDR-TB. The patients received the optimized background regimens (OBR) plus 100 mg (161 patients), 200 mg (160 patients), or placebo (160 patients) of DLM twice daily for 8 weeks. Sputum-culture conversion (SCC) was defined as a series of five or more sequential cultures negative for MTB. Accordingly, DLM was accompanied by an increase in SCC at 2 months among patients infected with MDR-TB. This finding emphasizes DLM as a treatment option for MDR-TB (Gler et al., 2012). In another clinical trial study (trial 208) reported by Skripconoka et al., treatment with DLM at two doses (100 and 200 mg twice daily) was continued in 421 patients for 6 months. In that study, 192/421 patients received DLM in combination with an OBR for ≥6 months, compared with 229/421 cases that received DLM for ≤2 months. In both trial studies (trials 204 and 208), sputum smear was negative in patients receiving DLM in comparison with patients receiving placebo or an OBR. However, the mortality rate decreased to 1% in 74.5% of patients who received DLM for 6 months, while this rate reduced by 8.3% in those who received the drug for 2 months. A favorable outcome was ultimately observed in patients with XDR-TB who consumed DLM (Skripconoka et al., 2013).

A former study inconsistently explored no association of DLM with an increase in SCC among patients infected with MDR-TB over 6 months. That research reported the results of phase three trial of DLM treatment in patients with pulmonary TB who received an OBR in Europe, Asia, Africa, and South America. Moreover, patients with MDR-TB were treated with either oral DLM at a dose of 100 mg twice daily for 2 months, followed by 200 mg once daily for 4 months (226 patients) or placebo with the same regimen (101 patients). Results of the same study showed that the difference in the median time for SCC between the two groups was insignificant during 6 months (von Groote-Bidlingmaier et al., 2019). Contradictory outcomes in the abovementioned surveys necessitate further investigation of DLM to determine its efficacy in SCC of MDR-TB (Szumowski and Lynch, 2015).

Tuberculosis (TB) is caused by various MTBC members such as MTB *sensu stricto* and *M. africanum*, as the main causes of TB in humans (Senghore et al., 2020). MAs, long-chain α-alkyl, and β-hydroxy fatty acids vary in size and structure and are indisputably necessary for the survival of MTBC arising in the cell envelope (Yimer et al., 2020). Although the lipidomics of MAs is very different among MTBC strains, the biosynthesis pathway of these fatty acids is equal (Portevin et al., 2014; Yimer et al., 2020). Mutations in the genes of the F420 signaling pathway of the MTBC, including *dnn*, *fgd1*, *fbiA*, *fbiB*, *fbiC*, and *fbiD*, can lead to DLM resistance (Reichmuth et al., 2020). Delamanid, as a broadly used anti-TB agent, exerted a greater *in vitro* antibacterial activity than other drugs, such as pretomanid (PTM), against MDR- and XDR-TB strains (Jing et al., 2019).

Delamanid (DLM) normally exists as a prodrug and is activated by a nitroreductase encoded by deazaflavin (F420)-dependent nitroreductase (*ddn*) gene in MTBC. This agent is cleaved into two reactive nitrogen species, including nitric oxide and nitrous acid with dual anti-TB effects via the disruption of the MA biosynthesis pathway, which is essential to the growth, survival, and disruption of the respiratory activity (Reichmuth et al., 2020).

The anti-TB activity of DLM has been examined in the cell culture system, wherein the intracellular activity against MTB is the main objective. The killing efficiency of DLM was similar to RIF, a reliable key antibiotic for intracellular MTB (Szumowski and Lynch, 2015). DLM is able to substantially inhibit the biosynthesis of cell wall during the disruption of the MA biosynthesis pathway, eventually giving rise to the elimination of MTB (Rustomjee and Zumla, 2015). Preclinical results have signified that DLM is effective against growing or dormant bacilli, both *in vitro* and *in vivo*. *M. bovi*s is a member of MTBC cultured under aerobic and anaerobic conditions to evaluate its killing capability in growing and dormant phenotypes. The *in vitro* results uncovered that *M. bovis*, the same as MTB, is susceptible to 0.016 mg/L of DLM in a growing state, and the dormant phenotype is more resistant to DLM (0.04 mg/L) (Szumowski and Lynch, 2015). In a guinea pig model of TB, the effective administration dose of DLM daily was 100 mg/kg to kill MTB within hypoxic lesions of the lungs (Chen et al., 2017). There were also several resistance-conferring mutations in *ddn* gene found in MTB and *M. bovis*, and DLM activation was abrogated during *ddn* mutation (Reichmuth et al., 2020).

#### Activity Against Non-tuberculous Mycobacteria

Despite the fact that MTB is the main cause of pulmonary disease (PD), the incidence and mortality of non-tuberculous mycobacterial pulmonary disease (NTM-PD) are escalating worldwide (Kim et al., 2019). The most common pathogens for NTM-PD are *Mycobacterium avium* (*M. avium*) complex (MAC), *Mycobacterium abscessus*, and *Mycobacterium kansasii* (Koh, 2017). According to a report by Krieger et al., DLM showed a wide variation in MIC against MAC and *Mycobacterium intracellulare* (0.013 to 0.4 μg ml^–1^). This result is the first evidence showing that DLM is a potential agent for the treatment of diseases caused by *M. avium* and *M. intracellulare* (Krieger et al., 2016).

The MIC of DLM against NTM is very variable. Soni et al. implied that the MICs of DLM against NTM were greatly different (0.1 to > 100 μg ml^–1^), and DLM indicated a higher MIC against only *M. kansasii* (0.1 μg mL^–1^) compared with other tested NTMs (Koh, 2017). Kim et al., however, denoted that MTB had high MIC values against NTM, except for *M. kansasii* (Kim et al., 2019). Recently, Yu et al. have confirmed the highly variable antimicrobial activity of DLM against NTM and reported that DLM has a high MIC against slowly growing *Mycobacterium* species of NTM, i.e., *M. avium* and *M. kansasii* (Yu et al., 2019).

## Delamanid Resistance

### Mechanisms of Resistance

Drug-resistant TB is globally a challenge for the TB control program performance. As predicted by mathematical modeling, the incidence of MDR- and XDR-TB will raise dramatically in the forthcoming decades (Koch et al., 2018). In this situation, emerging resistance to new drugs can create more complicated conditions for the treatment of TB (Nguyen et al., 2020). Therefore, the comprehension of fundamental mechanisms of drug resistance is essential to develop strategies for the optimal consumption of new effective drugs.

Resistance to DLM can be induced by non-synonymous mutations in five genes (*ddn, fbiA, fbiB*, *fbiC*, and *fgd1*) whose products are main proteins and coenzymes for the biosynthesis and modification of F_420_ (Figure 2). The F_420_-dependent nitroreductase coded by *ddn* gene activates F_420_. The mentioned enzyme can convert bicyclic nitroimidazole drugs to intermediate metabolites and desnitro form of DLM. *FbiA* gene encodes a transferase that catalyzes the transfer of a phosphoenolpyruvate moiety to F_420_-0.

*FbiB*, however, encodes a γ-glutamyl ligase that is involved in the conversion of dehydro-F_420_-0 to form F_420_-0 and then catalyzes the GTP-dependent successive addition of multiple gamma-linked L-glutamates to F_420_-0. *FbiC* encodes a F_420_-0 synthase that catalyzes the formation of the F_420_ precursor F_420_-0 (Fujiwara et al., 2018; Rifat et al., 2020). Another recently cloned gene involved in the biosynthetic pathway for the cofactor F_420_ is *fbiD* (*Rv2983*), which synthesizes the phosphoenolpyruvate moiety for subsequent steps done by *fbiA* (Bashiri et al., 2019). Ultimately, G6PD coded by *fgd1* gene is necessary for the redox re-cycle of F_420_ and catalyzes the oxidation of glucose-6-phosphate to 6-phosphogluconolactone using coenzyme F_420_ as an electron acceptor (Fujiwara et al., 2018; Hameed et al., 2018).

Thus, any mutations in this pathway result in the reduction of *Mycobacterium* bacilli to metabolize prodrug and low- to high-level DLM resistance. The prompt development of resistance to DLM highlights the necessity for the optimal employment of new drugs, stringent stewardship for drug resistance surveillance, and thorough awareness of drug resistance mechanisms. Therefore, supplementary studies can help understand the genetic and phenotypic changes attributed to clinically relevant DLM resistance, in order to establish rapid drug susceptibility testing methods (Li et al., 2019).

### Epidemiology of Resistance

A few studies have implied the DLM resistance in MTB isolates (Table 1). The spontaneous frequency of DLM resistance has been reported as 6.44 × 10^–6^ to 4.19 × 10^–5^, highlighting the importance of this drug in combination with other active anti-TB drugs during therapy (Szumowski and Lynch, 2015). A systematic review of mutations related to anti-TB drug resistance has stated 12 distinct mutations in the genes *fbiA, fbiC*, *ddn*, and *fgd1* associated with DLM resistance. The most frequent gene mutation investigated in the literature was *ddn* (*Rv3547*) W88, which increases MIC > 200 times (Kadura et al., 2020). Fujiwara et al. disclosed that mutations in each of these five genes led to the low metabolizing potency of *M. bovis* BCG Tokyo and MTB H37Rv mutants *in vitro*.

**TABLE 1:** Epidemiology of delamanid resistance.

References	Country	Published time	No. of MTB isolates	No. of DLM resistant isolates	MIC (μg/ml)	Mechanism of resistance	Resistance rate
Bloemberg et al., 2015	Switzerland	2015	1 (pre-XDR-MTB strain)	1	NM	Mutations in *fbiA* and *fgd1* genes	−
Hoffmann et al., 2016	Germany	2016	1 (XDR-MTB strain)	1	> 2.0	Mutations in *fbiA* gene	−
Schena et al., 2016	Italy	2016	19 (MTB clinical strains)	5	3 strains with > 32 and 2 strains with ≥ 1	Mutations in *ddn* and *fbiA* genes	21%
Stinson et al., 2016	United States	2016	460 (MDR-and XDR-MTB strains)	2 (MDR TB)	1 strain with 1 strain and 1 strain with > 8	Mutations in *ddn* gene	0.43%
Pang et al., 2017	China	2017	90 (all XDR-MTB strains)	4	1 strain with 0.5 and 3 strains with 32	Mutation in *fbiC* gene	4.4%
Yang et al., 2018	Korea	2018	420 (171 MDR, 139 Pre-XDR, and 110 XDR-MTB strains)	41 (15 MDR-, 17 Pre-XDR, and 9 XDR-MTB strains)	0.2	Mutations in *fbiA* or *ddn* (Gly81Ser and Gly81Asp) genes	10%
Polsfuss et al., 2019	Germany	2019	1 (XDR-MTB strain)	1	0.25	Mutation in *ddn*	−
von Groote-Bidlingmaier et al., 2019	Multicenter	2019	511 (Pulmonary MDR-MTB strains)	6 (Baseline resistance was reported in 2 of 511 and 4 of 341 participants during treatment (for 6 months)	NM	NM	At baseline 0.39% (2/511) and during treatment 1.17% (4/341)
Yu et al., 2019	China	2019	52 (33 RGM and 19 SGM NTM strains)	31 RGM and 8 SGM strains	> 0.25	Mutation in *ddn* gene	94% of RGM and 42% of SGM
Jing et al., 2019	China	2019	220 (110 MDR- and 110 XDR-MTB strains)	7 (4 MDR and 3 XDR-TB)	0.2	Mutation in *fbiA* (Glu249Lys) and *fgd1* (Phe320Phe) genes	3.18%
Battaglia et al., 2020	Multi-center	2020	124	39 (26 were resistant and 13 were low level of resistance)	≥ 0.12	Mutation in *ddn*, *fgd*, *fbiA*, *fbiB*, and *fbiC* genes	31.5% (21% resistant and 10.5% low-level resistance)
Kardan-Yamchi et al., 2020	Iran	2020	35 (all MDR-MTB strains)	9	> 0.125	Mutation in *ddn*, *fbiA*, and *fbiC* genes	25.7%
Reichmuth et al., 2020	Multicenter	2020	129	4	> 0.015	Mutations in ddn, *fbiC* (Ala416Val, Trp678Gly) and *fgd1* (Arg64Ser, T960C) genes	3.3%
Yoshiyama et al., 2020	Japan	2020	1 (MDR-MTB strain)	1	> 2.0	Mutation in *fbiC* gene	−
Wang et al., 2021	China	2021	391 (269 MDR-MTB,94 pre-XDR-MTB, 28 XDR-MTB strains)	13	NM	NM	3.32%
Zheng et al., 2021	China	2021	88 (all MDR-MTB strains)	4	> 0.125	Mutation in *fibC* and *ddn* genes	4.5%

*MTB; *Mycobacterium tuberculosis*; DLM, delamanid; NTM, non-tuberculous mycobacteria; RGM, rapidly growing mycobacteria; SGM, slowly growing mycobacteria; NM, not mentioned.*

They estimated that the *in vitro* spontaneous resistance frequencies for DLM ranged from 4.19 × 10^–5^ to 6.44 × 10^–6^ for MTB H37Rv and from 2.51 × 10^–5^ to 3.95 × 10^–5^ for *M. bovis* BCG Tokyo. This frequency of mutation is similar to INH and PTM mutation rates in laboratory conditions (Fujiwara et al., 2018). In another study, genotypic analysis revealed 11 new genetic polymorphisms in resistant strains compared with isolates recovered before starting DLM therapy. The two mutations in *fbiA* (D49Y and R175H) coincided with the development of phenotypic resistance to this new drug (Hoffmann et al., 2016).

Schena et al. evaluated DLM susceptibility testing of 194 MTB strains recovered from patients. By using resazurin microtiter assay (REMA), they identified four resistant isolates with MIC values of >32.0 mg/L. The genetic analysis of DLM resistance phenotypes illustrated four stop codon mutations in the *ddn* (Trp-88 → STOP) and *fbiA* (Lys-250→STOP) genes, leading to high MIC values in these strains (Schena et al., 2016). The finding of an *in vitro* drug susceptibility assessment of 90 clinical XDR MTB isolates in China indicated that four (4.4%) isolates increased MIC for DLM, and codon 318 mutation of *fbiC* gene was recognized as a novel mutation contributed to DLM resistance. The nucleotide sequence analysis of the other four genes related to DLM resistance showed no mutation in *ddn, fgd1, fbiA*, and *fbiB* genes. It seems that mutations in *fbiC* gene have a considerable role in DLM resistance, though further resistance mechanisms could not be neglected (Pang et al., 2017).

In 2018, Yang et al. investigated the MIC distributions of DLM in 420 clinical MTB strains in Korea. Considering the critical concentration of 0.2 mg/L, DLM resistance phenotypes were found in 41 strains (9.76%). Moreover, 31 out of 41 resistant strains harbored mutations in *fbiA* or *ddn* genes, and two non-silent mutations (Gly81Ser and Gly81Asp) were identified in *ddn* gene among the resistant strains. Following the observation of the Gly81Ser mutation in high frequency (75.6% in resistant strains and 81% in susceptible strains), the same authors interpreted that the importance of this mutation in DLM resistance is unclear (Yang et al., 2018).

In another evaluation of the *in vitro* activity of DLM accomplished by Jing et al. in China, among 220 clinical strains with no previous exposure to DLM, 3.18% of isolates were resistant. They attributed the detected unprecedented mutation in the *fbiA* gene (Glu249Lys) to high-level resistance to DLM and PTM in MTB, though no report of cross-resistance between DLM and PTM has been recorded to date (Jing et al., 2019).

In a multicenter study conducted from 2010 to 2019 in Korea, 633 patients with MDR-TB were analyzed. According to the results of MICs for DLM, three (11.5%) patients were considered resistant with interim critical concentrations of 0.016 mg/L. Among the three DLM patients resistant to TB, one had never been administrated anti-TB drugs in the past, and the remaining two patients had a history of only first-line anti-TB drug treatments (Lee et al., 2021). In a randomized phase III trial carried out in seven countries, 341 patients with MDR-TB received DLM-containing regimens for 6 months (DLM group), and 170 participants received placebo plus an OBR (placebo group). The rates of DLM baseline resistance and acquired DLM resistance in the DLM group were 0.39% (2/511 patients) and 1.17% (4/341 patients), respectively. However, no resistance to DLM was observed in the placebo group (von Groote-Bidlingmaier et al., 2019).

It is important to note that the addition of DLM to the therapeutic regimen of MDR- and XDR-TB patients should be based on individual evaluation, including drug susceptibility information, safety and tolerability of drug, and assessment of risk–benefit ratio. Additionally, the clinical administration of DLM to patients needs to be concomitant with the establishment of a systematic and consistent drug susceptibility testing, in order to recognize the primary emergence of drug resistance and to prevent the transition of resistant isolates (Stinson et al., 2016).

## Synergism of Delamanid With Other Antituberculosis Drugs

Despite the use of a wide variety of potent antibiotics for TB, an effective treatment is a serious challenge in the TB-affected patients (Heidary and Nasiri, 2016). Thus, the synergistic combination effect of drugs is regarded as an innovative approach to control TB infections (Ramón-García et al., 2011). The new anti-TB drug, DLM, is used to treat MDR- and XDR-TB. Several studies assessed the synergistic combination effect of DLM and BDQ and reached the conclusion that these drugs have a synergistic effect on INH- and RIF-monoresistant and also XDR-MTB clinical isolates (Ferlazzo et al., 2018; Pontali et al., 2018; Chandramohan et al., 2019; Olayanju et al., 2020).

The REMA suggested that the MICs (μg/ml) of DLM were 0.063 (alone) and 0.015 (combination) for INH-monoresistant, 0.063 (alone) and 0.015 (combination) for RIF-monoresistant, and 0.125 (alone) and 0.015 (combination) for XDR isolates. However, the MICs of BDQ were 0.25 (alone) and 0.015 (combination) for INH-monoresistant, 0.125 (alone) and 0.015 (combination) for RIF-monoresistant, and 0.063 (alone) and 0.015 (combination) for XDR isolates. Fractional inhibitory concentration indices (FICI) was also calculated using REMA-based calorimetric checkerboard assay (FICI ≤ 0.5 defined as synergy) to evaluate the synergistic effect of DLM. The obtained FICI for INH-monoresistant, RIF-monoresistant, and XDR isolates were 0.31, 0.30, and 0.37, respectively (Chandramohan et al., 2019).

In a prospective study, the effects of a single-drug regimen (BDQ) were compared with those of combination-drug regimen (BDQ-DLM) in South African patients with drug-resistant TB. The results revealed that the combination group possessed high prolongation of the QTcF (> 60 ms from baseline or >450 ms during treatment), but the long-term safety was greater for the combination than for the single-drug regimen (Olayanju et al., 2020). In this respect, the WHO recommended clinicians for using these antibiotics under specific conditions and not in combination, owing to their high risk for cardiotoxicity. In exceptional circumstances, clinicians may be obligated to combine these drugs; for instance, when the number of effective drugs (at least four drugs for an efficient regimen or in cases with few treatment options) is insufficient, as well as in difficult-to-treat cases or in the emergence of excessive XDR-TB resistance.

Ferlazzo et al., in a retrospective cohort study, described the early safety and efficacy of BDQ-DLM combination regimen, i.e., 100 mg of DLM twice a day and BDQ at a dose of 400 mg once a day for 2 weeks, followed by 200 mg of BDQ three times a week. That study was performed on 28 patients with drug-resistant TB in South Africa, Armenia, and India. Ferlazzo and associates observed no additive or synergistic QTc prolongation effect on BDQ-DLM regimen. It should be noted that no published data has yet reported the combined use of these drugs in children (Matteelli et al., 2015; Migliori et al., 2017; Ferlazzo et al., 2018; Pontali et al., 2018). In another retrospective cohort study conducted in Mumbai, India, treatment outcomes on 49 (70%) patients who received BDQ-DLM combination regimen for 24 weeks were represented to be successful. The majority (69%) of patients showed culture conversion, and effective end-of-treatment results were reported in 49 (70%) patients (Das et al., 2020a).

An *in vitro* and *in vivo* study of DLM exhibited low MICs (in the range of 0.006–0.024 μg/ml) and low doses for an effective therapeutic activity on MDR-TB. In addition, DLM was more effective in intracellular MTB compared with RIF (at the concentrations of 3 and 0.1 μg/ml, respectively). In another investigation, a combination regimen, comprising of DLM (2.5 mg/kg), RIF (5 mg/kg), and pyrazinamide (PYR; 100 mg/kg), known as synergistic combination drugs, was employed for the treatment of mice (*n* = 6) (Matsumoto et al., 2006).

The results of that study indicated faster eradication (by at least 2 months) and shorter duration of clinical treatment of viable TB in the lungs of murine compared with the standard regimen, i.e., RIF (5 mg/kg), INH (10 mg/kg), ETB (100 mg/kg), and PYR (100 mg/kg) (Matsumoto et al., 2006). Given that new cephalosporins possess a broad spectrum of antibacterial activities, it is likely that these antibiotics have high efficiency for TB therapy. In this respect, *in vitro* synergy assays have suggested strong synergistic interaction between β-lactams (cephradine and faropenem) and DLM (FICI of ≤0.5) for MDR- and XDR-TB, which might possibly be effectual in reducing treatment length. Hence, an *in vivo* study could validate the synergistic effect of DLM on β-lactams (Ramón-García et al., 2016).

Since the substitution of moxifloxacin for the first-line drug regimen may be more effective in MDR-TB and DLM has a substantial impact on MDR- and XDR-TB, a combination of these drugs could serve as an effective synergistic combinatorial therapy. In this context, Chandramohan et al. conducted a study focusing on the *in vitro* synergistic effect of DLM on moxifloxacin against drug-resistant clinical MTB isolates (INH- and RIF-monoresistant, MDR, Pre-XDR, and XDR-MTB. The MICs of the drug were evaluated by the REMA, and REMA-based calorimetric checkerboard assay was carried out to assess the FICIs (FICI ≤ 0.5 defined as synergy). The synergistic effect was exhibited against INH- and RIF-monoresistant and XDR isolates with FICI of 0.5, 0.5, and 0.24, respectively (Chandramohan et al., 2019).

The MICs (μg/ml) of DLM and BDQ were, respectively, 0.063 and 0.25 for INH-monoresistant, 0.063 and 0.125 for RIF-monoresistant, and 0.125 and 0.063 for XDR isolates, when used alone, while the MIC of the two drugs in combination was 0.015 for all the isolates. However, WHO reported that the combination of moxifloxacin and DLM might increase the risk of QT prolongation (World Health Organization, 2016). Additionally, owing to the high rate of moxifloxacin resistance, using a combination of these drugs for treating XDR-TB was inappropriate in clinical settings. Thus, an *in vivo* study is highly needed to validate the toxicity, efficacy, and safety of these drugs (Chandramohan et al., 2019).

There is an abundant room for future progress in determining the particular characteristics of pharmacokinetics and drug safety of new regimen, including DLM, for vulnerable populations such as pregnant women, children, and patients with HIV (Lienhardt et al., 2020). In 2016, the WHO issued an interim guideline for administrating DLM in 6- to 17-year-old children. However, evidence on the safety and efficacy of DLM treatment in children is limited. One of the largest cohorts of children and adolescents treated with new anti-TB regimens was carried out in India. For 24 patients (aged 0–19 years) with fluoroquinolone (FQ)-resistant TB, a treatment regimen, including DLM and BDQ in combination and separately, was utilized between September 2014 and June 2018. The results of final treatment outcomes and culture conversion showed that the treatment was successful in 96% of patients, and only 2/12 cases had side effects during treatment with the new TB drugs (Das et al., 2020b).

In a study conducted by Pieterman et al. (2021), the synergistic effect of combination regimen (DLM (2.5 mg/kg), BDQ (25 mg/kg), and linezolid (100 mg/kg) was compared with the standard regimen (ETB, PYR, RIF, and INH) in TB-infected mice. Their results confirmed that the combination regimen is more effective than the standard regimen. One reason for this superiority was culture negativity in the lungs, which was observed at 8 weeks in combination regimen vs. 20 weeks in the standard regimen. Another reason was that in the combination regimen, relapse was found only in one mouse, while in the standard regimen, it was continued at 24 weeks. Consequently, in view of very limited clinical studies, further investigations are necessary to determine the synergistic combination effect of DLM on other drugs, and more *in vivo* surveys are required to corroborate the synergistic effect of DLM and to determine their toxicity, efficacy, and safety.

## Pharmacokinetics and Pharmacodynamics

**FIGURE 3:**
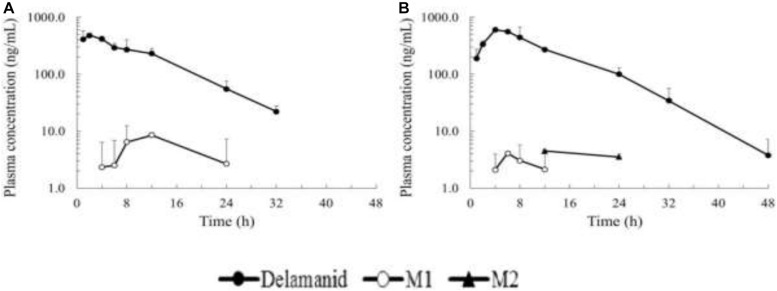
Plasma concentration time profiles of delamanid single oral dose and M1/M2 metabolites in mice **(A)** and rats **(B)**.

After catalyzing to the human-unique metabolite M, which is formed by the cleavage of the 6-nitro-2,3-dihydroimidazo[2,1-b]oxazole moiety, DLM was metabolized by three individual pathways. Hydroxylation of the oxazole moiety of M1, the most crucial starting point, to form M2, and also consecutive oxidation to the ketone form (M3), are two main pathways in humans that are mostly performed by CYP3A4 (Figure 3) (Sasahara et al., 2015).

For anti-TB compounds, there are various mechanisms of interindividual PK variability and drug–drug interactions. One of these mechanisms is the induction of metabolism or its inhibition through hepatic cytochrome p450 enzymes. Another mechanism includes drug transportation by P-glycoprotein, a significant efflux transporter that affects intracellular pharmacokinetics by transporting foreign substances out of the cells. In preclinical surveys, DLM is recognized as highly protein bound (> 97%), and its metabolism is mainly mediated by plasma albumin. However, the hepatic CYP enzymes, particularly CYP3A4, CYP1A1, CYP2D6, and CYP2E1, likely show a lesser extent. In animal (e.g., dog, rat, and mouse) models, DLM has been explored to have an oral bioavailability of 35%–60% (Miyamoto et al., 2005; Lewis and Sloan, 2015).

Using fresh human hepatocytes and human liver microsomes, *in vitro* investigations studied the effect of DLM on human cytochrome p450 enzymes. DLM did not show any effect on CYP1A1/2, CYP2A6, CYP2B6, CYP2C8/9, CYP2C19, CYP2D6, CYP2E1, and CYP3A4 at concentrations <100 μmol/L (Matsumoto et al., 2006). In healthy volunteers, an interaction was observed between DLM and the strong CYP3A4 enzyme inducer, RIF, which diminished exposure to DLM by 47%. This reductive behavior could be the main reason that the coadministration of DLM with the mentioned CYP enzyme inducers was contraindicated by the European Medicines Agency. *In vitro* evidence revealed that clinically relevant interactions of DLM with drugs, particularly those whose disposition is dependent on ATP-binding cassette (ABC) and solute carrier transporters, breast cancer-resistant proteins (BCRP/ABCG2), organic anion-transporting polypeptides, or organic cation transporter 1, are impossible (Hu et al., 2016).

The complete oral bioavailability of DLM has not yet been measured; however, it seems to be between 25 and 47%. During dose-escalation studies, administration of higher oral doses was associated with a less than proportional increase in plasma exposure. Exposure to DLM, unlike a number of first-line anti-TB drugs (especially RIF), was enhanced by food, i.e., high-fat meal in particular. Exposure in a fed state is almost three times higher than that of fasted state. In combination regimens, varied absorption profiles between drugs may make coadministration complicated. DLM, a high percentage (> 99%) protein bound, has an exceptional apparent volume of distribution (Oye et al., 2013; Chang and Sotgiu, 2017).

The elimination of DLM is performed directly from plasma, but not urine, with a half-life of between 30 and 38 h. Its metabolization is also thought to be carried out, to a great extent, by plasma albumin. Although the full metabolic profile of DLM is unknown, this drug is seemingly converted to its primary metabolite, DM-6705, following the reaction of amino groups in serum albumin to this agent. Thereafter, the DM-6705 is broken down by hydrolysis and CYP3A4 and converted to some other metabolites, which concentration raises to steady state during 6–10 weeks. These metabolites often show weak anti-TB activity, whereas other metabolites may indicate DLM-related toxicity. For instance, the DM-6705 metabolite has a key function in the prolongation of QTc. However, the metabolic pathway of DLM appeals for further investigation to fully be elucidated (Oye et al., 2013; Lewis and Sloan, 2015).

## Adverse Events

Apart from many beneficial impacts of DLM on MDR- and XDR-TB, the side effects of its use have been reported in some patients. Research of genotoxicity and carcinogenic potential indicates no serious impact on human and animals (Lewis and Sloan, 2015; Hanaki et al., 2017). Although the concern about hepatotoxicity of DLM is not as much as other anti-TB drugs such as RIF, INH, and PYR, it has been suggested that DLM may rarely have the potential effects on the liver.

Recent surveys have demonstrated the potential of DLM and/or its metabolites on cardiac repolarization, which resulted in increased QTcF prolongation (Lewis and Sloan, 2015). Because of QTc-prolonging effects on several second-line MDR-TB drugs, including FQs, co-administration of clofazimine and BDQ with DLM increases the concerns about cardiac arrhythmias (Matsumoto et al., 2006). It is speculated that the increased QTc prolongation is associated with the main DLM metabolite DM-6705 (Deltyba Epar Product Information, 2014). It has also been recommended that in patients with hypoalbuminemia (level\2.8 g/dL), DLM therapy should not be administrated due to the associated increased risk of QTc prolongation (Ryan and Lo, 2014). Other frequent drug adverse events, including gastrointestinal problems (vomiting, decreased appetite, gastritis, and nausea), tremor, asthenia, and hypokalemia, were reported in patients who received DLM (Chang and Sotgiu, 2017). In a study conducted in Addis Ababa from 2017 to 2019, 51 drug-resistant TB patients were registered. Thirty (58.8%) patients developed side effects, the most common of which were gastrointestinal and hematological disorders and QTc prolongation. Also, 20 patients permanently discontinued the offending drug (Tesema et al., 2021).

In a multinational phase II trial, 204 patients received an OBR combined with DLM or placebo for 2 months. A comparison between the DLM and placebo groups showed that the prevalence of QTc interval prolongation in patients who received DLM was significantly higher than that in the placebo group. However, no clinical symptoms, such as arrhythmias or syncope, were observed in patients with the episodes of QT interval prolongation (Gler et al., 2012). More investigation was performed for patients who completed trial 204, and DLM was administrated twice daily together with an OBR for an additional 24-month period. In that observational study, serious adverse events happened in 11.7% of the DLM-treated group, and the most prevalent adverse events reported were anemia, hemoptysis, QTc interval prolongation, and psychotic disorder (Skripconoka et al., 2013).

The Korea Centers for Disease Control and Prevention Agency reviewed 318 patients who were administrated with BDQ and/or DLM for 6 months, to evaluate treatment outcomes. They reported two patients with QTcF prolongation (QTcF interval 506 and 511 ms) and five cases with gastrointestinal problems during treatment with DLM. The physician decided to permanently discontinue DLM due to adverse events for all seven patients (Kang et al., 2020). A pediatric clinical trial determined the safety and pharmacokinetics of DLM for patients with MDR-TB. Among 19 enrolled patients, a 17-year-old patient with XDR-TB had experienced renal impairment, severe vomiting, and severe electrolyte disorders, and subsequently QTcF prolongation. After temporary discontinuation of treatment and the management of electrolyte imbalance and vomiting, the therapy was re-established (Tadolini et al., 2016). In another retrospective cohort study in South Korea, the safety and tolerability of DLM in 32 enrolled patients were assessed. Three patients exhibited increased QTcF prolongation (QTcF interval of >500 ms) during treatment with DLM. Therapy with this drug re-initiated at 7–10 days after the transient discontinuation of treatment (Mok et al., 2018).

Lee et al. published an analysis of medical records of 129 patients who treated for MDR-TB from January 2005 through December 2017 in South Korea. Regardless of the most frequent side effects related to Linezolid, an increased QTcF prolongation was observed in one patient with FQ-sensitive MDR-TB during treatment with DLM (Lee et al., 2019). Hughes et al. followed up 58 patients with RIF-resistant TB to determine the adverse effects of DLM treatment program in South Africa. The most common side effects related to DLM consumption were vomiting, QTcB > 450 ms, and myalgia. Moreover, the gradually worsening QT interval prolongation and cardiac symptoms, including chest pain, dizziness, and palpitations, were observed in one patient (Hughes et al., 2019).

Mok et al. retrospectively reviewed 49 patients who received DLM and had final treatment outcomes after 24 weeks. They described four patients, of whom three cases died due to sudden cardiac problems, pneumonia, and acute myocardial infarction at 1 to 3 months following the completion of DLM therapy. Besides, the remaining one patient experienced an increased QTcF interval at 4 weeks after the administration of DLM (Mok et al., 2019). It was recommended that before the beginning and throughout the period of treatment with DLM, frequent monitoring of electrocardiograms is essential. In patients with QTcF > 500 ms, DLM treatment either should not be initiated or should be interrupted. Moreover, the serum electrolytes are required to be assessed at baseline, and in patients with electrolyte disturbances, especially hypokalemia and hypocalcemia, DLM should not be administrated (Deltyba Epar Product Information, 2014).

## Clinical Trials

Although European Medicines Agency’s Committee for Medicinal Products for Human Use approved the DLM 8 years ago for use in a combination therapy for pulmonary MDR-TB cases, there are a few clinical studies investigating the exact role of DLM in the treatment of MDR- and XDR-TB (Lohrasbi et al., 2018). Gler et al., in a randomized controlled trial (RCT), presented the outcomes for 481 HIV-negative patients with pulmonary MDR-TB who received oral DLM at higher (200 mg twice daily) or lower (100 mg twice daily) doses, or placebo for 8 weeks plus a background drug regimen developed based on WHO guidelines.

The sputum of each patient was cultured weekly in Lowenstein–Jensen medium and in a mycobacterial growth indicator tube (MGIT) system, and ≥5 successive weekly cultures negative for MTB were defined as culture conversion. The results of both Lowenstein–Jensen and MGIT cultures demonstrated that the patients who received 100 or 200 mg of DLM twice daily had a significantly higher rate of culture conversion compared with those who received placebo. The analysis of adverse events in that clinical trial showed that the patients who received 100 mg of DLM had fewer adverse events than those who received 200 mg; most of the events were of similar incidence to patients who received placebo. While the groups receiving DLM had more frequent QT prolongation relative to the placebo group, no episodes of a prolonged QT were associated with clinical events. Overall, the outcomes of that investigation recognized DLM as a promising treatment options for MDR-TB (Gler et al., 2012).

In another double-blind phase three RCT, which was performed by Von Groote-Bidlingmaier and colleagues, the safety and efficacy of DLM for pulmonary MDR-TB therapy were investigated (von Groote-Bidlingmaier et al., 2019). A total of 511 patients received DLM at a dose of 100 mg twice daily for 8 weeks, followed by 200 mg once daily for 16 weeks or placebo plus an OBR, developed based on WHO guidelines. The sputum of patients were cultured weekly in the MGIT system, and the results showed no difference in mean time to culture conversion between DLM and placebo groups. Safety assessments indicated that 98% of patients had at least one clinical manifestation, of which approximately 27% were serious. None of the fatal adverse events were considered to be related to DLM. The outcomes of that study demonstrated no clinically significant episodes of a prolonged QT and provided evidence of the safety profile of DLM to support its use with other QT-prolonging antibiotics (such as BDQ) for MDR-TB therapy (von Groote-Bidlingmaier et al., 2019).

Recently, Dooley et al. conducted a phase two RCT on the effects of DLM and/or BDQ on QT interval in patients with drug-resistant TB. They enrolled 84 cases with MDR-TB or RIF-resistant TB taking multidrug background therapy. Clofazimine was not allowed, and levofloxacin replaced moxifloxacin. The patients received DLM at a dose of 100 mg twice daily for 6 months and BDQ at a dose of 400 mg daily for 2 weeks, followed by 200 mg three times weekly for 5.5 months. The results of electrocardiograms showed that the median QTc change from baseline was 8.6 ms (DLM), 12.3 ms (BDQ), and 20.7 ms (DLM + BDQ). Therefore, a QTc interval of DLM + BDQ was not more than additive. In addition, no participants had a grade 3 or 4 QTc manifestation, and no deaths occurred during the study. These outcomes provide supportive evidence of the safety of simultaneous DLM and BDQ use in patients with MDR-TB or RIF-resistant TB (Dooley et al., 2021).

Mallikaarjun et al. carried out a phase 1 RCT to investigate the interactions between DLM and other anti-TB antibiotics, including ETB and Rifater (RIF + INH + pyrazinamide) in healthy individuals. In that study, 55 individuals received multiple oral doses of DLM + placebo or DLM + ETB-Rifater or placebo + ETB-Rifater once daily. Plasma samples were then analyzed for DLM and its metabolites by liquid chromatography-tandem mass spectrometry assay. The results showed that the coadministration of DLM with other anti-TB antibiotics has no clinically significant interactions (Mallikaarjun et al., 2016). In another phase 2 RCT, Zhang et al. performed a study to indicate the clinical benefit of DLM during MDR-TB therapy (Zhang et al., 2013).

In their survey, 38 HIV-negative patients with pulmonary MDR-TB assigned and received 100 or 200 mg of DLM twice daily or placebo for 8 weeks in combination with an OBR, including six anti-TB agents for MDR-TB therapy. During 10 weeks, culture conversion occurred among 32 patients. The safety analysis indicated clinical manifestations, including QT prolongation, hypercortisolemia, and psychiatric adverse events, among 13 cases. Taken together, their evaluation demonstrated that DLM was effective and well tolerated for MDR-TB treatment (Zhang et al., 2013).

In another study conducted by Koirala et al., the effectiveness of BDQ (and/or DLM) containing regimens in TB patients was evaluated. The antibiotic resistance pattern of the patients was severe (> 30% with XDR-TB). The median number of resistant drugs was 6 (Blair and Scott, 2015; Diel et al., 2015; Diel et al., 2015; Cox et al., 2018; World Health Organization, 2020; Khoshnood et al., 2021) and 5 (Blair and Scott, 2015; Cox et al., 2018; Heidary et al., 2019; Khoshnood et al., 2021) in patients with a final outcome and overall cohort, respectively. The proportion of patients achieving culture conversion and sputum smear ranged from 88.8 to 89.3% (patients with a final outcome) to 92.8% and 93.4% (whole cohort), respectively. Among the patients treated with BDQ, but not DLM, 284 (74.2%) achieved treatment success. However, 63 (16.5%) patients were lost to follow-up, 11 (2.9%) failed, and 25 (6.5%) died (Koirala et al., 2021).

## Conclusion

The increasing global prevalence of MDR- and XDR-TB highlights an urgent need for more effective drugs. Unfortunately, a few studies have hitherto addressed the effect of DLM on drug-resistant MTB. Available evidence suggests that due to the low resistance of DLM among MDR and XDR strains of MTB, this antibiotic can be considered as a promising anti-TB drug. Moreover, the synergism of DLM in combination with BDQ can ameliorate the effectiveness of this agent against pan-drug-resistant MTB isolates.

## Author Contributions

All authors listed have made a substantial, direct and intellectual contribution to the work, and approved it for publication.

## Conflict of Interest

The authors declare that the research was conducted in the absence of any commercial or financial relationships that could be construed as a potential conflict of interest.

## Publisher’s Note

All claims expressed in this article are solely those of the authors and do not necessarily represent those of their affiliated organizations, or those of the publisher, the editors and the reviewers. Any product that may be evaluated in this article, or claim that may be made by its manufacturer, is not guaranteed or endorsed by the publisher.
